# Frameless stereotactic body radiation therapy for multiple lung metastases

**DOI:** 10.1120/jacmp.v15i4.4737

**Published:** 2014-07-08

**Authors:** Qilin Li, Jinming Mu, Wendong Gu, Yuan Chen, Zhonghua Ning, Jianxue Jin, Honglei Pei

**Affiliations:** ^1^ Department of Radiation Oncology The Third Affiliated Hospital of Soochow University, The First People's Hospital of Changzhou City Jiangsu Province China; ^2^ Department of Radiation Physics Elekta China Co. Ltd. Beijing China

**Keywords:** multiple lung metastases, frameless stereotactic body radiation therapy, interdigitation‐capable multileaf collimator, four‐dimensional cone‐beam CT, two‐step plan

## Abstract

Two patients with multiple lung metastases (≥ 5) were treated using frameless stereotactic body radiation therapy (SBRT) on an Elekta Axesse linear accelerator equipped with an interdigitation‐capable multileaf collimator and four‐dimensional cone‐beam CT (4D CBCT). The technique and the early clinical outcomes were evaluated. Patient A with five lung metastases and Patient B with seven lung metastases underwent SBRT (48 Gy/8 fractions for Patient A, 42 Gy/7 fractions for Patient B). The treatments were administered using a 6 MV photon beam. The nominal dose rate was 660 MUs/min. Patients were positioned and immobilized using thermoplastic masks and image guidance was done using 4D CBCT. The targets were delineated on the images of the 4D CT, and the positron emission tomography‐computed tomography (PET‐CT) images were taken as references. A two‐step, volumetric‐modulated arc therapy (VMAT) plan was designed for each patient. Step 1: the lesions in one lung were irradiated by a 210° arc field; Step 2: the rest of the lesions in the other lung were irradiated by a 120° arc field. Plans were evaluated using conformity index (CI) and homogeneity index (HI). Patients were followed up and adverse events were graded according to the Common Terminology Criteria for Adverse Events v4.0 (CTCAE v4.0). The beam‐on time of each treatment was less than 10 min. The CI and HI for the two plans were 0.562, 0.0709 and 0.513, 0.0794, respectively. Pulmonary function deteriorated slightly in both patients, and the patient with seven lung lesions was confirmed to have Grade 1 radiation pneumonitis. The technique was fast, accurate, and well tolerated by patients, and the two‐step plan is a helpful design in reducing the dose to the lungs.

PACS numbers: 87.55‐x, 87.56.J‐, 87.56.‐v, 87.56.nk, 87.57.qp

## I. INTRODUCTION

Stereotactic body radiation therapy (SBRT) is a technique for delivering large doses to tumors and is characterized by highly conformal dose distributions.^1^, ^2^ In recent years, SBRT has been shown to be an effective treatment option for inoperable patients with lung cancer and metastatic lung lesions. Noticeable local tumor control rates had been reported for both.^1^, ^2^, ^3^, ^4^ However, most reports in this area documented patients with only one lung lesion.^2^, ^4^, ^5^ The studies carried out on patients with multiple lung lesions were rare. The study by Kelly et al.^(6)^ involved patients with up to three metastatic lung lesions and reported no cases of grade 4 or 5 toxicity. Okunieff et al.^(3)^ had done a study involving patients with more than five metastases, but the number of lung metastases in one patient was unclear, and the details of the treatment technique were not disclosed. There are no reports which explicitly state the outcomes of SBRT in patients with five or more lung lesions.

The delivery systems for SBRT include multiple coplanar and/or noncoplanar beams, volumetric modulated arcs using a linear accelerator, helical tomotherapy or the CyberKnife system.^2^, ^5^, ^7^ All systems must have motion management and image guidance capabilities. For these reasons, a fixed three‐dimensional coordinate system and a stereotactic body frame are important components.^1^, ^8^ but are usually invasive and generally painful. Frameless image‐guided SBRT has been explored in several studies recently.^9^, ^10^, ^11^ In all of these, SBRT was used with cone‐beam computer tomography (CBCT) and included the six degree of freedom (6 DOF) image registration and alignment technique. One of the most up‐to‐date technologies, four‐dimensional (4D) CBCT, has been applied to radiation treatment of patients with lung cancer.^12^, ^13^

Axesse (Elekta AB, Stockholm, Sweden) is one of the latest high‐end linear accelerators made by the company. It is equipped with Agility, a high‐definition interdigitation‐capable multileaf collimator (IC‐MLC, 160 leaves with a width of 5 mm at isocenter) (Table 1). The image guidance system is comprised of 4D CBCT and XVI software (version 4.5, Elekta AB), and a robotic 6 DOF patient positioning system (6 DOF treatment couch HexaPOD with iGuide Software Version 1.1, Medical Intelligence, Schwabmünchen, Germany). This type of accelerator has the capability of implementing frameless SBRT. One Axesse was fully commissioned and put into use in our hospital in August 2012.

In this study, two patients with five and seven lung metastases, respectively, underwent frameless SBRT with Axesse. The feasibility and early clinical outcomes of this treatment were evaluated.

**Table 1:** Parameters of the multileaf collimator of Axesse (Agility)

*Parameter*	*Value*
Field size maximum	400 mm
Leaf individual travel range (with respect to DLG)	200 mm
Leaf interdigitation range	200 mm
Leaf and DLG combined travel range	350 mm
Diaphragm/jaw overtravel relative to central axis	120 mm
Diaphragm/jaw speed, maximum	90 mm/s
Leaf speed, maximum	35 mm/s
Leaf and DLG combined speed, maximum	65 mm/s

DLG=dynamic leaf guides; all leaves were integrated with two DLGs and they traveled together.

## II. MATERIALS AND METHODS

### A. Patients

This study was approved by the Institutional Review Board of the First People's Hospital of Changzhou (the Third Affiliated Hospital of Soochow University) and written informed consent was obtained from the patients before treatment. Two male patients with multiple lung metastases were selected. One patient (A) was a 64‐year‐old man with five lung metastases from liver cancer, the other (B) was a 57‐year‐old with seven lung metastases from esophageal cancer. Both patients had received several kinds of therapy, including surgery, radiation therapy (RT), and chemotherapy. The primary lesions had been locally controlled. For both patients, the lung nodules appeared in both lungs on computer tomography (CT) scans in the first months of 2012, but the disease progressed quickly a few months later when assessed with a positron emission tomography‐computed tomography (PET‐CT). The Karnofsky Performance Status (KPS) were 80 (Patient A) and 90 (Patient B), respectively.

### B. Radiotherapy planning and SBRT

**Figure 1:**
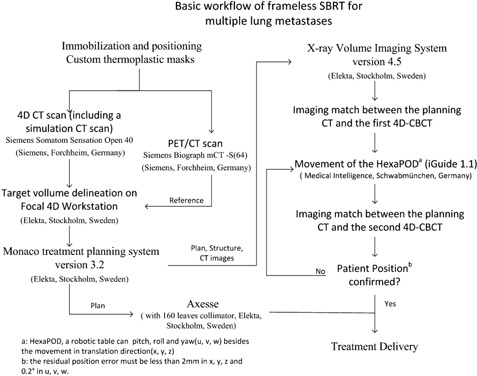
The workflow of frameless SBRT

**Figure 2:**
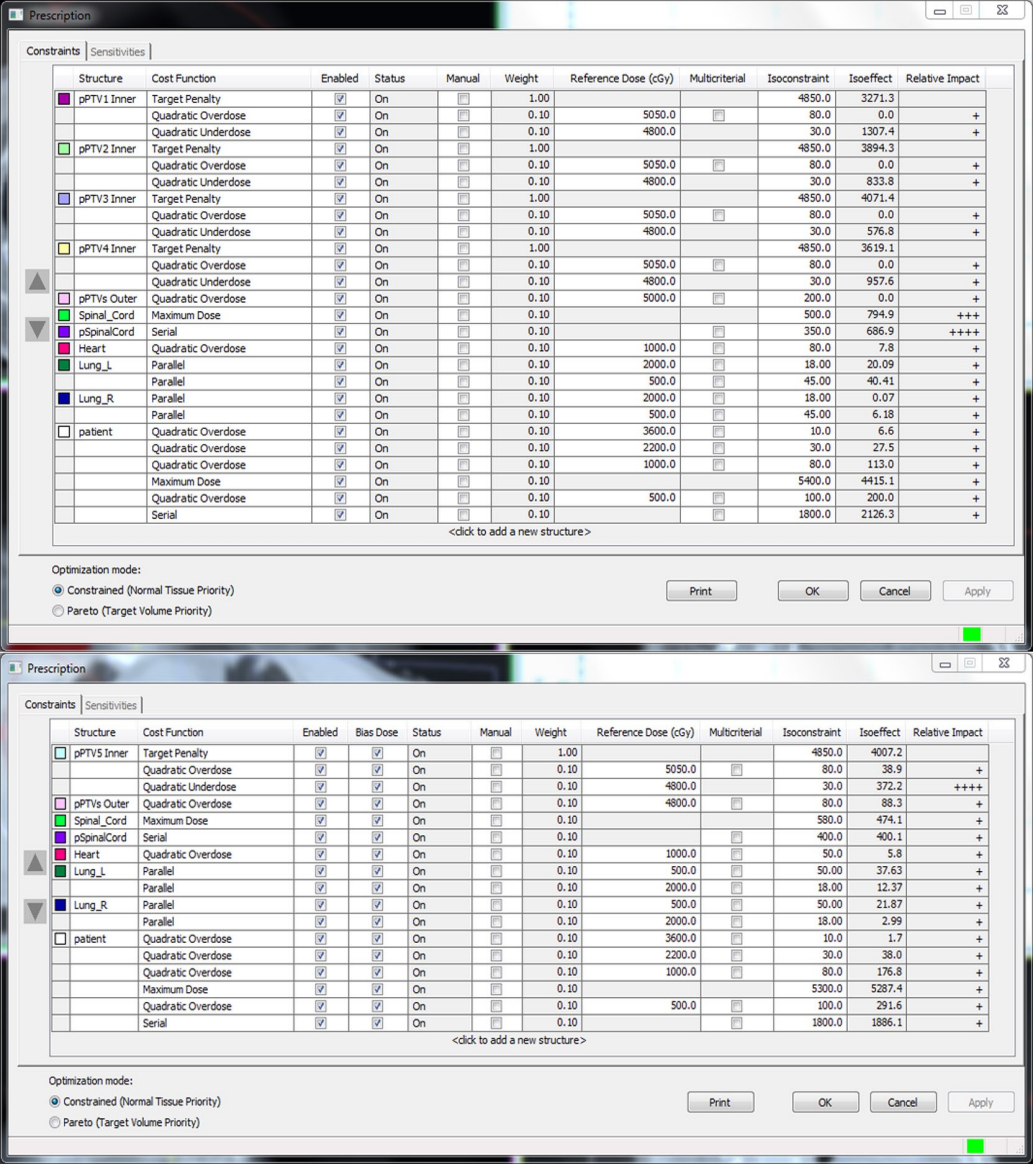
The upper part shows the parameters of the first step, and the lower part those of the second step. The “pPTVn Inner” was created by expanding PTVn by 2 mm radially. The “pPTVs Outer” was created by expanding all PTVn by 1 cm radially. Spinal cord was expanded by 5 mm to create the “pSpinalCord”. The column “bias dose” indicates that the dose in the first step is taken into account in the second step.

**Figure 3:**
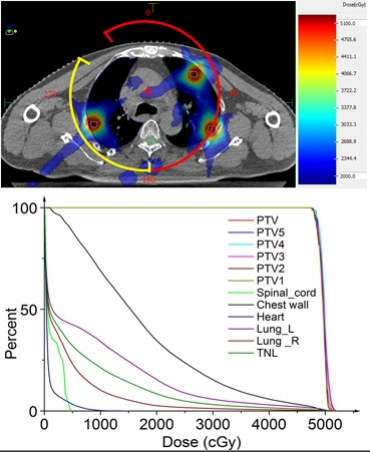
(Upper) The dose distribution on one traverse slice (Patient A with five metastases). The red and yellow arcs represent the fields; the red “+” is the isocenter of the fields; the cyan outlines represent the targets on this slice. (Lower) The plan DVH for Patient A. PTVn=the different lung targets, PTV was the sum of all PTVn; TNL=total normal lungs, meaning the volume (left lung+right lung–CTV); CTV=clinical target volume.

The treatment workflow is shown in Fig. 1. Patients underwent PET/CT scans and the images were taken as reference for delineating the internal tumor volumes (ITV). The delineation of the ITV was done using the “maximum intensity projection” on a Focal 4D workstation. The ITV was then expanded by 2 mm radially and 3 mm craniocaudally to create the planning target volume (PTV). The treatment planning system (TPS) was Monaco 3.2 (CMS Software Inc., St Louis, MO), in which the fast X‐ray voxel Monte Carlo algorithm^14^, ^15^, ^16^ was used in the final dose calculation. The prescriptions for patient A and B were 48 Gy/8 fractions, 42 Gy/7 fractions, respectively. The dose was prescribed to cover 95% of the PTV. The treatments were administered using a 6 MV photon beam and delivered every other day. The nominal dose rate was 660 MUs/min. For both patients, metastases were found in both the right and left lung. In order to lower the doses delivered to both lungs, a two‐step plan was designed for each patient. Step 1: the lesions in one lung were irradiated by a 210° arc field (the lesions in the other lung were not included in the prescription of this step); Step 2: the rest of the lesions in the other lung were irradiated by a 120° arc field (based on the dose contribution of Step 1, like a boost plan). The parameters of the two‐step plan for Patient A are exhibited in Fig. 2. The two fields shared one isocenter (Fig. 3). Treatment plans with only a 360° arc beam were done for comparison (Table 2).

**Table 2:** Comparison of two types of treatment plans

				*PTV*			*Lungs*			*Spinal Cord*	*Heart*		*Chest Wall*	
	*Plan Type*	*CI*	*HI*	*D99(cGy)*	*D95(cGy)*	*D2(cGy)*	*V5(%)*	*V20(%)*	*Dmean(cGy)*	*Dmax(cGy)*	*Dmean(cGy)*	*D2(cGy)*	*V30*	*D8cc(cGy)*
Patient A	Partial Arcs	0.562	0.0709	4789.1	4839.9	5134.6	32.1	8.46	578	481.3	73.7	622.5	58cc	4760.3
	Whole Arc	0.583	0.073	4759.8	4802.3	5139.6	37.52	10.85	679.2	559.1	78	527.6	59.3cc	4782.5
Patient B	Partial Arcs	0.513	0.0794	4145.4	4215.7	4488.6	46.98	14.39	891.4	594.3	723.2	1683.3	37.1cc	3760
	Whole Arc	0.533	0.0769	4151.3	4210.4	4478	54.61	16.63	997.6	688.9	939.5	1910.3	39.2cc	3815.6

Dx=the dose to x%volume of tissue, as displayed on the DVH, unit: cGy; Vx=percentage of tissue receiving x Gy of radiation, unit: %; Dmax, mean: the max, mean dose to the tissue, unit: cGy; V30=how much volume of chest wall received 30Gy; D8cc=the dose to 8cc volume of chest wall, as displayed on the DVH, unit: (cGy); D8cc=a statistically significant risk factor for the development of rib fracture.^(25)^

### C. 4D cone‐beam CT and image matching

**Figure 4:**
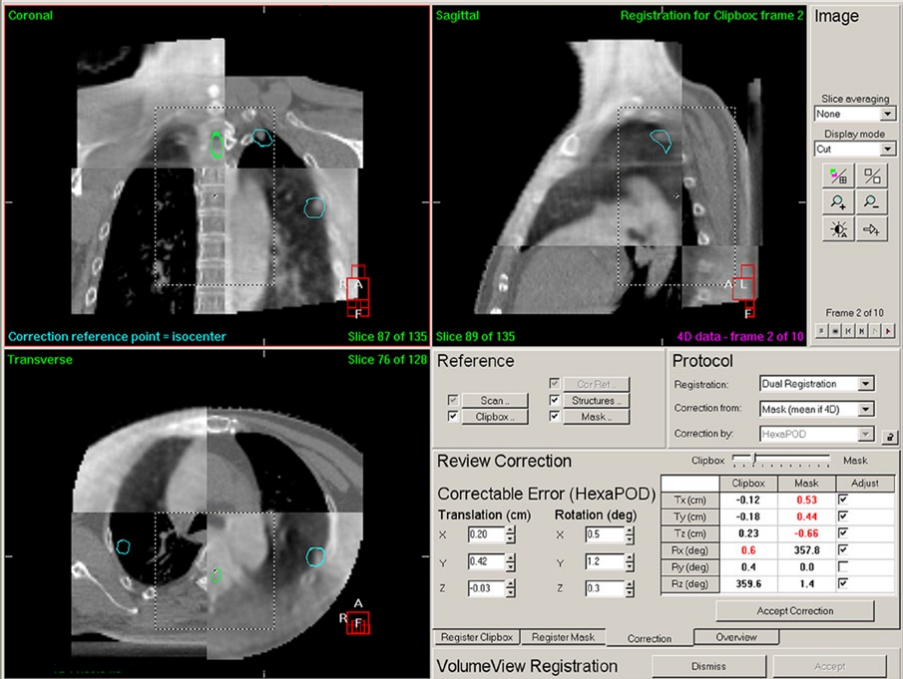
The transverse, sagittal, coronal slices of the 4D CBCT and the planning CT and the resulting image matching are shown. The display was quartered. Dual registration was used. The couch was a HexaPOD (6 DOF). Mask (mean if 4D) meant the correction based on the mask area on the average of all phases. Move the slider between Clipbox and Mask, the display of 4D CBCT and the numbers in the table under the slider would change accordingly. So does the correctable error.

There was a direct correlation between the diaphragm motion and respiration. The respiratory signal can be extracted from the 2D projection data during a CBCT scan^(17)^ to reconstruct 4D CBCT images. In this study, the respiration cycle was divided into ten phases. The planning CT (3 mm slice thickness) was a simulation CT scan done just before the 4D CT scan. After the 4D CBCT reconstruction, image matching between the 4D CBCT and the planning CT was carried out. The workflow was described below (Fig. 4).

**Figure 5:**
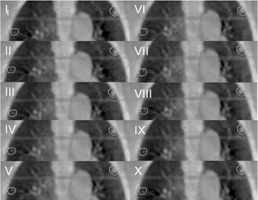
The result of image matching between the planning CT and ten phases of 4D CBCT (Patient A). The contours represent the PTV.

Dual registration (clipbox and mask) was used in this study. The image matching was mainly done with automatic registration and the method used was grey value matching. The clipbox is the rectangular area with a dashed line, and the mask is the area of the PTV expanded by 5 mm radially. The registration was based on the content in the clipbox or the mask. The first step was the clipbox registration. The images were quartered, half of which were 4D CBCT and the rest were planning CT scan images. The cut point could be moved by the mouse so that the differences between these two were easily observed. When the matching was finished, the 10 phases of 4D CBCT were shown animatedly. The differences were checked in each phase. The next step was the mask registration. The third step was to review the corrections (the lower right part of Fig. 4). Based on the results of the registrations, the correctable error could be determined only from the clipbox registration or the mask registration, or the compromise between them. When the slider between the clipbox and the mask moved, the figures, the numbers in the table under the slider, and the correctable error were changed accordingly. The results of the image matching were checked carefully in each phase of 4D CBCT. When all 10 phases of the 4D CBCT coincided with the corresponding slices of the planning CT, and the moving tumor was seen to stay in the PTV contour (Fig. 5, and Appendix A), the button “Accept correction” was clicked, and the correctable error sent to the HexaPOD patient positioning system.

The image matching was performed twice before each treatment delivery to ensure that the PTV coverage was adequate in all directions, throughout the whole respiratory cycle. The time for each 4D CBCT was about 240 s, and for the registration about 120 s every time. Although the image matching was a little time‐consuming, it was worthwhile and necessary.

### D. Plan quality

Target coverage, sparing of organs at risk (OAR), conformity index (CI), and homogeneity index (HI) were used to evaluate the plans. CI was defined as follows:^(18)^

(1) $$CI=\frac{Vol_{T\arg et}}{Vol_{Rx}}\times 100\%$$

${\text{Vol}}_{\text{Target}}$ and ${\text{Vol}}_{\text{Rx}}$ represent the volume of the target (e.g., PTV) and the target volume irradiated receiving the prescription dose. The ideal CI for a plan is 100%. The higher the CI, the better the plan.

The HI was defined as follows:^(18)^

(2) $$HI=\frac{D_{2}-D_{98}}{D_{Rx}}\times 100\%$$

$D_{2}$ and $D_{98}$ describe the doses to 2% and 98% of the target volume, respectively, as displayed on the dose‐volume histogram (DVH), and represent the maximum and minimum doses received by the tissue. $D_{\text{Rx}}$ stands for the prescription dose to the target. The HI of a good plan should be as small as possible.

### E. Follow‐up and toxicities

Patients were followed up with clinical examinations and CT imaging every two months for six months after the treatment, and thereafter every six months.

The results at the first follow‐up are shown in the next section. All the tumors in both patients were peripherally located. Rates of local control and severity of radiation pneumonitis were of the primary endpoints. Radiation pneumonitis was graded according to the Common Terminology Criteria for Adverse Events v4.0 (CTCEA v4.0). Other potential toxicities, including chest wall pain and rib fracture, were monitored and recorded.

## III. RESULTS

Table 2 shows the dose characteristics, HI, and CI of the two kinds of treatment plans. The HI, CI, and the dose to chest wall were nearly the same in all plans, but lung V20 and V5 (Vx: percentage of tissue receiving x Gy of radiation) in the two‐step plans were significantly lower than those in the whole arc plans, especially V5. The doses delivered to spinal cord and heart were rather low in all plans. The specific plan information including fields, MUs, beam‐on, and CBCT scan times are exhibited in Table 3. The beam‐on time for each treatment delivery was less than 10 min. But the 4D CBCT and image matching took some time (the overall treatment time was then around 25 min). The results of the pulmonary function tests for both patients, before and two months after SBRT, are shown in Table 4, and indicated that pulmonary function in both patients deteriorated slightly. Dose distributions in one transverse CT slice, the arc fields, and DVHs of the plan for Patient A are shown in Fig. 3.

All the lesions almost disappeared. Grade 1 radiation pneumonitis was found in Patient B. The results of imaging matching of the 4D CBCT and the planning CT are shown in Fig. 5 (see also Appendix A video SV1, which shows that the PTV coverage are adequate during the whole respiratory cycle.).

**Table 3:** Basic SBRT plan information for Patients A and B

	*Prescription (Gy/fx)*	*PTV Volume (cc)*	*The First Step*			*The Second Step*			*CBCT Scan Time(s)*	
			*Arc (CCW)*	*MU*	*Time(s)* ^a^	*Arc (CCW)*	*MU*	*Time(s)* ^a^	*3D CBCT*	*4D CBCT*
Patient A	48/8	15.89	180/330	3172.3	355	300/180	1678.9	147	120	240
Patient B	42/7	23.52	30/180	3205.2	359	180/60	1057.9	138	120	240

a The nominal dose rate: 660 MUs/min.

CCW=counterclockwise;fx=fraction.

**Table 4:** Pulmonary function tests for patients before and two months after SBRT

	*Before RT*			*Two Months After RT*		
	*SVC/%Pred*	*FVC/%Pred*	FEV1.0/%Pred	*SVC/%Pred*	*FVC/%Pred*	*FEV1.0/%Pred*
Patient A	2.82L/79.7	2.03L/57.3	1.72L/60.6	2.59L/73.2	1.85L/52.3	1.59L/56
Patient B	3.05L/88.2	2.88L/83.2	2.59L/94.9	2.94L/85	2.79L/80.6	2.53L/92.7

SVC=static vital capacity; FEV1.0=forced expiratory volume in 1 s; FVC=forced vital capacity; %Pred=percentage of the predicted value; RT=radiation therapy.

## IV. DISCUSSION

Patients with lung metastases often have more than one lesion. It is a well‐known fact that conformal radiation therapy (CRT) for multiple metastases is extremely difficult, both from the treatment planning and the implementation points of view. For these patients there are rarely effective therapeutic options, and though chemotherapy is always a possibility, outcomes are generally disappointing.^(19)^

With the fast developments in radiotherapy treatment hardware and software during the last decade, two techniques have become available for the treatment of patients with multiple metastases. The first is helical tomotherapy. The study by Sterzing et al.^(7)^ has already shown that helical tomotherapy is capable of treating multiple lesions. However, the machines are available only in some cancer centers.

The other technique uses linear accelerators equipped with IC‐MLC such as the Varian Trilogy and TrueBeam with Millennium MLC (52, 80, or 120 leaves with different width; Varian, Palo Alto, CA),^(20)^ the Elekta Synergy with MLCi2 (80 leaves with a 10 mm width at isocenter), and the Elekta Axesse with Agility (160 leaves with a 5 mm width at isocenter; Elekta AB).^(21)^ These accelerators have the ability to shape beams to produce multiple “dose islands”, which is a relative requirement for patients with multiple lesions. This technique makes it possible to irradiate multiple targets simultaneously (see Appendix B video, SV2, which displays the moving multileaf collimator during beam‐on) and reduces the treatment time significantly. Timmerman et al.^(22)^ reported that on average 30 to 45 min was needed for each SBRT treatment of stage I non‐small cell lung cancer (NSCLC). Baba et al.^(23)^ reported that the irradiation time per fraction for patients with a single lung lesion was less than 30 min. Static beams were chosen in these two studies for the delivery of SBRT.

In our study, the beam‐on times per fraction for patients with multiple lesions were less than 10 min (Table 3). Had the treatment been delivered by an accelerator with an MLC without the capability of interdigitation, treatment time may have exceeded the time tolerated by patients. The interdigitation‐capable multileaf collimator should be essential for the radiation treatment of patients with multiple (≥3) lesions.

The main advantage of RT in this study was that the treatment was guided by 4D CBCT. 4D CBCT is a relatively new commercially available technique. The respiratory signal needed for 4D image reconstruction relates directly to the motion of the diaphragm and can be extracted from the 2D projection data obtained during the CBCT scan.^(17)^ The workflow using 4D CBCT in stereotactic volumetric‐modulated arc therapy (VMAT) for a lung tumor has been reported in detail by Nakagawa et al.^(12)^ The difference between that study and ours is that the treatment couch in our department is a 6 DoF HexaPOD (this couch can pitch, yaw, and roll, as well as the usual movement in the translational directions). The use of the HexaPOD had been described by Meyer et al.^(9)^ The rotational shifts used to correct patient position were very important in RT.^(11)^ In addition, 4D CBCT guided SBRT is more accurate than SBRT using 3D CBCT.^(13)^ Imaging matching the 4D CBCT and the planning CT of Patient A (Fig. 5) indicated that the PTV outline totally covered the range of the motion of the tumors during the whole respiratory cycle.

The main side effect of 4D CBCT was that the time needed for scanning and image matching was a little longer than that for 3D CBCT. In this study, the overall treatment time, including scanning and image matching two 4D CBCTs, was around 25 min. This procedure is still less than that in the studies by Timmerman et al.^(22)^ and Baba et al.^(23)^ This may be partly due to the fact that this technology is new to our department, but with increased use, the proficiency and the skills required for image matching will improve and thereby shorten the procedure time.

In this study, two patients with multiple lung metastases underwent SBRT guided by 4D CBCT. Radiation pneumonitis (RP) was the major complication of concern (all tumors were peripherally located). In the RTOG 0813 protocol,^(8)^ it was required that the percentage of total lung volume receiving 20 Gy (lung V20) should be less than 10%, and lung V20 in the range of 10% to15% was classified as minor violation of the protocol. The V20 in our patients was 8.46% and 14.39%, respectively (Table 2).

Compared to the plans using one 360° arc field, the two‐step treatment plans decreased the value of lung V5 (the percentage of total lung volume receiving 5 Gy) 5.42 percentage points for Patient A and 7.63 for Patient B (Table 2). It has been reported by several studies^24^, ^25^, ^26^ that lung V5 was significantly associated with the incidence of pulmonary complications. The design of the two‐step plan is a valuable help in reducing dose to the lungs, and helps to lower the incidence of RP.

Both patients had previously received external beam RT (EBRT). There was some overlap with the previous fields in both patients. The reirradiation was a cause for concern. However, patients with peripheral tumors are generally less likely to experience severe toxicity after SBRT.^(27)^ Kelly et al.^(6)^ reported that there were no cases of Grade 4 or 5 toxicity in patients who underwent lung SBRT after prior EBRT to the thorax. Two months after SBRT, Patient B with seven lesions was confirmed to have Grade 1 RP and pulmonary function in the two patients degraded slightly (Table 4).

Chest wall pain and rib fracture are also of concern in patients with peripheral lung lesions treated with SBRT. However, Kim et al.^(28)^ reported that the median time to rib fracture for patients receiving SBRT is 17 months. Neither of our patients experienced these toxicities in the 12 month follow‐up.

## V. CONCLUSIONS

This study focused primarily on the technique of frameless SBRT for multiple lung lesions treated with the Axesse linear accelerator combined with IC‐MLC and 4D CBCT. And the two‐step plan with partial arcs is a helpful design in reducing the dose to the lungs. This technique is feasible, well‐tolerated by patients, and resulted in good responses with minimal toxicity. The treatment is fast and accurate, and results in minimal patient discomfort both during and after therapy.

## ACKNOWLEDGMENTS

The authors thank the help from the physicists of Elekta Corporation for the technique support and their comments on the manuscript. This work received funding support from The Department of Health, Jiangsu Province, China (Grant Number: H201350). The funding organization played no role in the collection of data, its analysis and interpretation, and publication of this manuscript.

## APPENDICES

### Appendix A: Supplemental Video, SV1

### Appendix B: Supplemental Video, SV2

## Supporting information

Supplementary Material

Supplementary Material

Supplementary Material

Supplementary Material
